# Effect of plant fibers on the physical properties of slurry-processed reconstituted tobacco

**DOI:** 10.3389/fchem.2024.1463648

**Published:** 2024-11-20

**Authors:** Tong Liu, Yixuan Wang, Chunping Wang, Qi Zhang, Le Wang, Yuhan Li, Linyang Xu, Xinyan Jin, Xianzhong Yin, Zhan Zhang, Chong Luo, Lili Fu, Yangbing Wen, Bin Li

**Affiliations:** ^1^ Tianjin Key Laboratory of Pulp and Papermaking, Tianjin University of Science and Technology, Tianjin, China; ^2^ Zhengzhou Tobacco Research Institute of CNTC, Zhengzhou, China; ^3^ China Tobacco Henan Industrial Co., Ltd., Zhengzhou, China

**Keywords:** reconstituted tobacco, plant fibers, beating degree, mechanical properties, thermal conductivity

## Abstract

The primary function of plant fibers in reconstituted tobacco is to enhance the physical strength, and it can quite modify their physical properties. This study demonstrated the effect of various plant fibers and their beating degrees on the physical properties of reconstituted tobacco. Tensile index, burst index, uniformity, tensile stiffness orientation, and thermal conductivity coefficient were examined. The result revealed that the mechanical properties of reconstituted tobacco varied according to the type and beating degree of the fibers. The mechanical properties of softwood, cotton, and bast fibers showed an initial increase followed by a decrease with increasing beating degree, while bamboo fiber showed a continuous improvement in mechanical properties proportional to the beating degree. Conversely, hardwood fiber displayed an inverse relationship with its beating degree. Under identical beating conditions, reconstituted tobacco containing softwood fibers showed the greatest improvement in tensile properties, achieving the highest tensile strength, thermal conductivity, and specific heat capacity. In particular, when softwood fibers were beaten to 50 °SR, the physical properties of the reconstituted tobacco peaked, with longitudinal and transverse tensile indices improving by 42.48% and 12.11%, respectively. Additionally, the bursting resistance index increased by 61.93%, and the thermal conductivity coefficient increased by 5.94%.

## 1 Introduction

Reconstituted tobacco had become the primary raw material for heated tobacco products (HTPs) due to its low heated temperature and lack of combustion met the physiological needs of consumers, and substantially reduced the release of harmful ingredients (such as tar and carbon monoxide) (Davis and Nielsen, 1999; Wang et al., 2023; Zhang et al., 2024). The current prevalent production processes for reconstituted tobacco include four main technologies: paper-based, slurry-processed, rolled-compressing, and dry-formed reconstituted tobacco. Slurry-processed reconstituted tobacco (SPRT) showed excellent features, like high homogenisability, simple processing technology, low energy consumption, and high production efficiency (Sung et al., 2008). During the preparation of SPRT, tobacco powder, plant fibers, adhesives, and atomizing agents were uniformly mixed to form a slurry, which was subsequently degassed and dried, ensuring these components were evenly distributed. SPRT presented a compact structure, high thermal conductivity, and excellent processing stability, which made it an ideal raw material for HTPs (Gao and Chen, 2017; Li et al., 2018). The mechanical strength of the reconstituted tobacco affected its adaptability during the production process of HTPs, and its physical characteristics, such as excellent effective component loading rate and high thermal conductivity, were conducive to improving the quality of HTPs. The primary function of plant fibers in reconstituted tobacco is to enhance the physical strength, its degree and amount of processing directly impact the overall performance of reconstituted tobacco (Wu et al., 2014; Chuet al., 2024). Insufficient fiber addition can weaken the physical strength of the reconstituted tobacco, making the subsequent cutting and forming process difficult. Conversely, an excess of fibers can lead to fiber aggregation, which can disrupt the uniformity of the reconstituted tobacco and adversely affect the consuming experience by causing an unpleasant woody taste. Miao et al. (2022) found that the 45 °SR fibers significantly strengthened the calcium carbonate plate, compared to the unbeaten 8 °SR fibers, resulting in a 50% increase in flexural strength and a 160% increase in tensile strength. This paper aimed to systematically analyze the characteristic changes of different plant fibers treated with varying beating degrees, clarify the corresponding relationship between fiber structure, physical strength**,** and thermal conductivity of reconstituted tobacco, optimize the production process control of reconstituted tobacco leaves, and provide a scientific basis for solving problems in industrial production. The study offers novel insights into enhancing the quality of SPRT, thereby facilitating its practical applications in HTPs.

## 2 Materials and methods

### 2.1 Materials

Bleached softwood pulp boards, hardwood pulp boards, bast pulp boards, bamboo pulp boards, and cotton pulp boards were supplied by Tianjin Wood Elf Company, the cellulose content was more than 90%, while xanthan gum, atomizing agent, and tobacco powder were supplied by China Tobacco Henan Industrial Co. Ltd.

### 2.2 Disintegrating and beating of plant fibers

The plant fibers were separately configured with water to form the solution with 5% concentration and unclogged fibers by using the fluffer, and then the absolute-dry fibers, which were obtained after the solution was pumped and filtered, were configured as a mixture with 10% concentration. After that, this mixture was beaten using the PFI mill, and the beating degree of fibers was controlled by the clearance of the grinding disc and the number of machine revolutions. Finally, plant fibers of (20 ± 1)°SR, (40 ± 1)°SR, (50 ± 1)°SR, (60 ± 1)°SR, (80 ± 1)°SR were obtained.

### 2.3 Preparation of SPRT

A mixture containing tobacco powder, atomizing agent, xanthan gum, and plant fiber in a 100:20:3:3 ratio, with a concentration of 20%, would be uniformly mixed and placed in a vacuum degasser to remove gas. The resulting slurry would be poured into the casting machine trough at a speed of 0.15 m/min and heated to produce reconstituted tobacco at a rate of (130 ± 5) g/m^2^. The casting machine trough slit was set at 0.75 mm. Heating and drying modules with temperatures were conducted in the drying process of six different sections under temperatures of 80°C, 80°C, 70°C, 70°C, 65°C, and 60°C, respectively.

**FIGURE 1:**
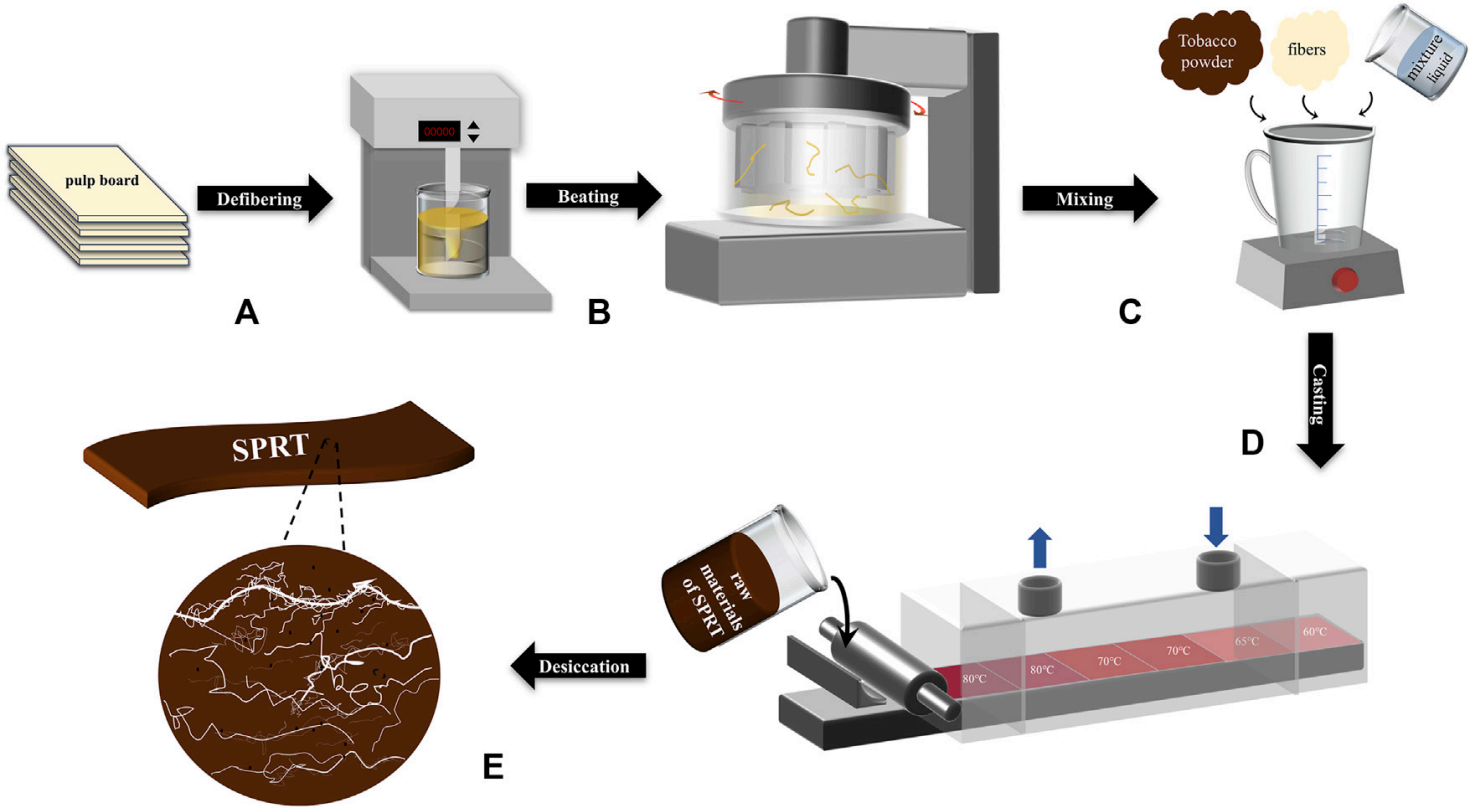
Flow chart of SPRT **(A)** defibering by the fluffer **(B)** beating by the PFI mill **(C)** mixing uniformity with the blender **(D)** tape casting by the casting machine **(E)** desiccation to get SPRT.

The process of fiber pretreatment and preparation of reconstituted tobacco in this work is shown in Figure 1.

### 2.4 Fiber morphology analysis and surface micro-morphology

Fiber length, size distribution, and percentage of fine fibers were determined using a fiber analyzer (L&W Fiber Analyzer).

Fiber samples were obtained by freeze-drying different plant fibers, and then the fibers were bonded to a conductive adhesive. Furthermore, the fibers were sprayed with gold for microscopic observation of the fibers using a scanning electron fiber microscope (SEM).

### 2.5 Characterization of mechanical properties of SPRT

The physical properties of the reconstituted tobacco were tested by placing it in a constant temperature and humidity box. The conditions were set at a temperature of (23 ± 1.0)°C and a relative humidity of (50 ± 1.0)% for 12 h to reach equilibrium. The physical properties (tensile, bulk, bursting index) of reconstituted tobacco samples were measured according to the ISO standards methods. For the tensile strength test, the slurry spread direction during the preparation of reconstituted tobacco was identified as longitudinal, and the vertical direction was determined as transverse. Subsequently, the longitudinal and transverse tensile strengths were tested.

Calculated the tensile index according to Formula 1:

Y=S/g×1000 (1)

in the formula, Y represents the tensile index, and the unit is N·m·g^-1^; S stands for tensile strength in KN·m^-1^; g stands for quantitative, in g·m^-2^。

Calculated the bulk according to Formula 2:

U=g/d (2)

in the formula, U represents the loose thickness, and the unit is cm^3^/g; d stands for thickness in cm.

### 2.6 Tensile stiffness orientation test for SPRT

The Tensile Stiffness Orientation Tester (150 TSO Tester, L&W) used ultrasonic measurement to assess paper stiffness in all directions, enabling rapid fiber orientation distribution analysis.

## 3 Results and discussion

### 3.1 Fibers morphology analysis

**FIGURE 2:**
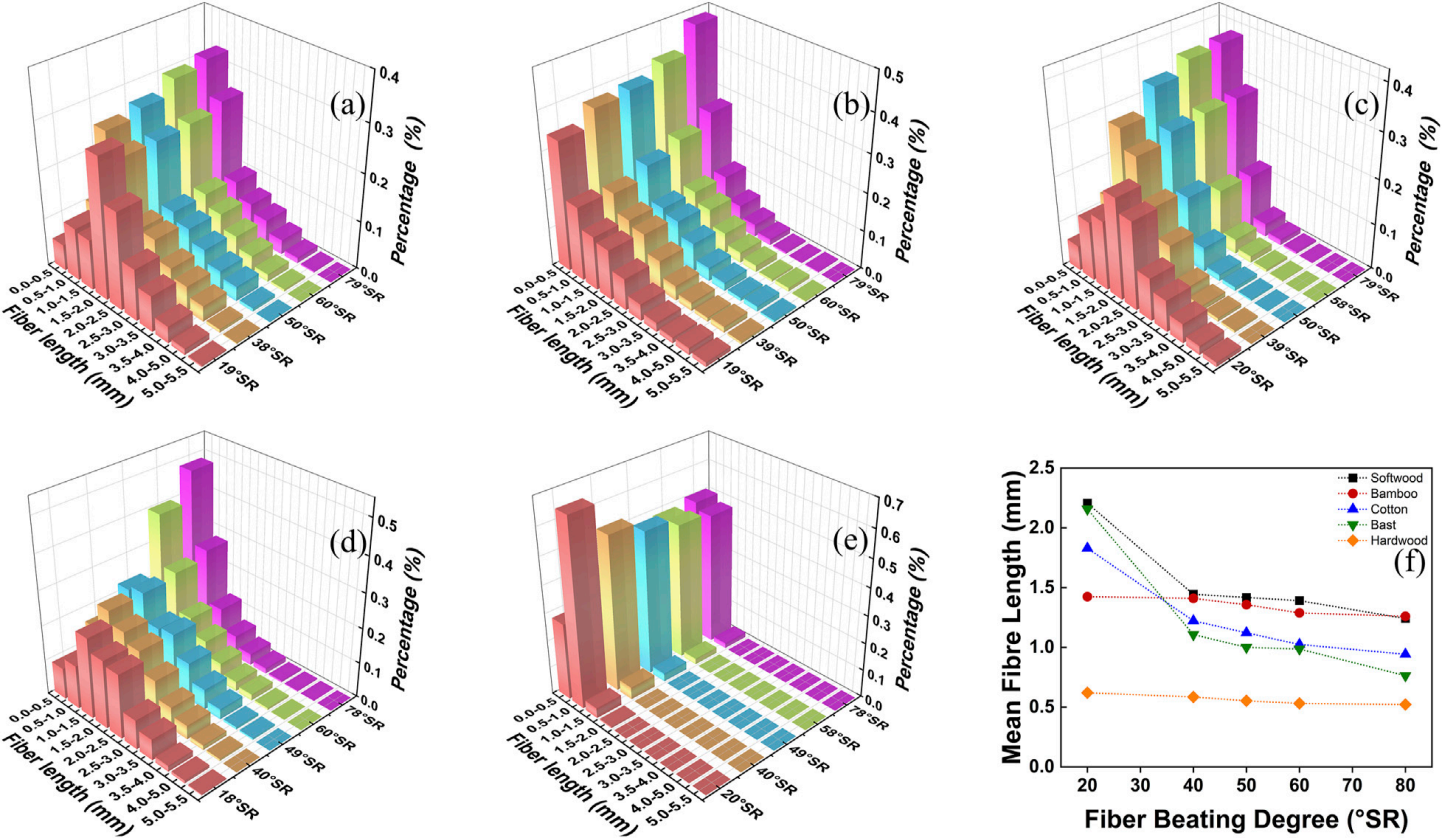
Effect of beating degree on length and size distribution of **(A)** softwood fibers **(B)** bamboo fibers **(C)** cotton fibers **(D)** bast fibers **(E)** hardwood fibers; **(F)** Effect of beating degree on the average length of different kinds of fibers.

The amount of plant fibers added to reconstituted tobacco using SPRT was relatively small. However, it had a significant impact on the length, diameter, degree of spinning, content of fine components, physical strength, and thermal conductivity of the reconstituted tobacco leaves (Prasad et al., 2004; Kim et al., 2017). The beater process not only enabled fibers to spin but also shortened the length of the fibers, altering their distribution in reconstituted tobacco. (Chen et al., 2011; Chen et al., 2013; Zhao et al., 2017; Du et al., 2022). In this study, we evaluated five common plant fibers: softwood, bamboo, cotton, bast, and hardwood. As shown in Figures 2A–F, while the beating degree increased from (20 ± 1) °SR to (80 ± 1) °SR, we observed significant changes in the proportion of fine fibers in softwood, cotton, and bast fibers (Gao et al., 2015). For instance, softwood fiber length in (0.0–0.05) mm increased from 0.055% to 0.134% after beating 79 °SR, additionally, the average fiber length almost decreased to about half of the original length, from 2.205 mm to 1.241 mm. The proportion of bamboo fibers and hardwood fibers with length distribution of (0.0–0.5) mm increased from 0.34% to 0.28%–0.48% and 0.50%, respectively, and the average fiber length decreased from 1.423 mm to 0.621 mm–1.259 mm and 0.523 mm, respectively (Figures 2B,E). Conversely, we observed minimal size variation in hardwood and bamboo fibers, meaning their size distribution and average length remained relatively consistent under the same beating degree (Sarrazin et al., 2009; Zhao et al., 2018; Axelrod et al., 2022; Dong et al., 2023).

**FIGURE 3:**
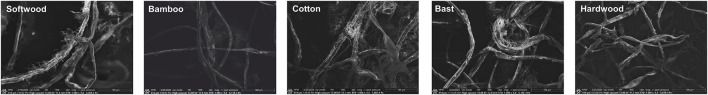
Scanning electron microscopy (SEM) of five different plant fibers at (50 ± 1) °SR.

Scanning electron microscopy (SEM) analysis in Figure 3 showed that when fibers were beaten, softwood, cotton, and bast fibers displayed the most noticeable surface fuzziness and spinning compared to hardwood and bamboo fibers. Hardwood and bamboo fibers were less affected by the beating process, and they exhibited lower degrees of spinning.

### 3.2 Analysis of the effect of plant fibers on the physical properties of SPRT

The reconstituted tobacco used for HTPs must meet specific post-processing requirements and have sufficient physical strength. Additionally, it must be able to generate and release aerosols when heated, with the heat transfer process being facilitated by a compact structure. In this context, this paper focused on studying the mechanical properties and thermal conductivity to ensure tobacco products meet the necessary criteria for subsequent processing and application (Zhao et al., 2018).

**FIGURE 4:**
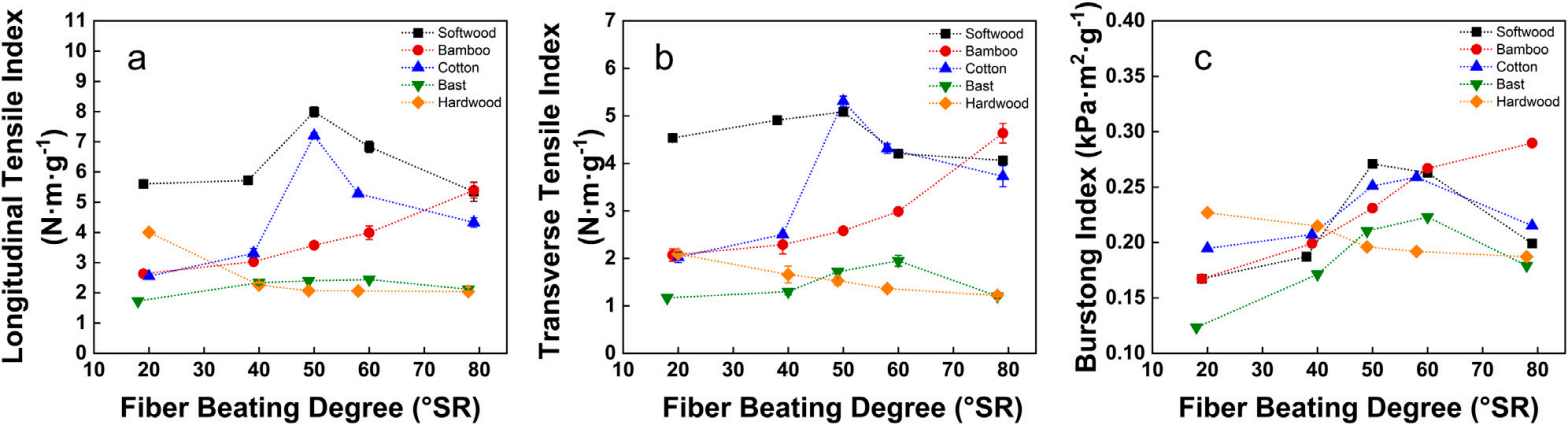
The longitudinal and transverse tensile index and bursting index of SPRT with different fibers added.

To illustrate our strategy, we conducted a proof of concept study using the longitudinal tensile index, transverse tensile index, and bursting resistance index as representatives to characterize the change rule of the mechanical properties of the reconstituted tobacco with the fibers beating degree (Figure 4). The results indicated that the longitudinal and transverse tensile index as well as bursting resistance index of reconstituted tobacco with softwood fibers, cotton fibers, or bast fibers first increased and then decreased with the beating degree from 20 to 50 °SR, which thus highlights the significant advantages of fibers could improve mechanical properties within this range. However, when the beating degree exceeded 50 °SR, the mechanical properties weakened for the three types of reconstituted tobacco. In this regard, the mechanical properties of reconstituted tobacco with bamboo fibers in the wake of the beating degree increased, on the contrary, those supplemented with hardwood fibers experienced a gradual decrease in mechanical properties as the beating degree increased (Gümüşkaya et al., 2006; Afra et al., 2013).

When the beating degree was around (50 ± 1) °SR, the tensile and bursting index of reconstituted tobacco with softwood fibers and cotton fibers reached their best values. The longitudinal tensile indexes were significantly raised to 7.992 N·m·g⁻^1^ and 7.203 N·m·g⁻^1^, which had 42.48% and 182.14% higher properties than those supplemented with (20 ± 1) °SR fibers, respectively. The transverse tensile indices were also improved to 5.082 N·m·g⁻^1^ and 5.309 N·m·g⁻^1^, which were 12.11% and 162.56% higher, respectively. Additionally, the bursting index saw an increase to 0.271 kPa·m^2^·g⁻^1^ and 0.259 kPa·m^2^·g⁻^1^, which were 61.93% and 28.87% higher, respectively. These results indicated that the beating degree significantly improved the impact of adding cotton fibers to the reconstituted tobacco (Dong et al., 2023).

As illustrated in the proof of principle studies, when softwood, cotton, and bast fibers were beaten to a moderate level, their size distribution was appropriate, with a suitable quantity of fine fibers. The beating degree increased the yarn-spinning, specific surface area, and exposure of active hydroxy-groups of the fibers, which enhanced the stability of the fibers network in reconstituted tobacco and improved their physical properties. However, excessive beating degree led to the results that produced an excess of fine fiber content and the fiber length was too short to provide physical properties, those all were not conducive to improving the physical properties of reconstituted tobacco. Those weakening effects were due to a reduction in the binding force between shortened fibers, which outweighed the strengthening effect brought by fibers spinning (Bhattarai et al., 2020; Dong et al., 2023). When reconstituted tobacco with the same beating degree, the mechanical properties were enhanced by the addition of softwood fibers compared to cotton fibers. This is because softwood fibers, obtained under similar beating conditions, are longer and harder than cotton fibers, with higher strength and toughness. The thicker cell wall of softwood fibers, containing more lignin, helps maintain rigidity and straightness during the beating process, on the other hand, cotton fibers are relatively shorter and softer, with higher cellulose and lower lignin content, and a natural spiral twisting structure, which aids in interweaving between fibers (Wang et al., 2024).

When the beating degree of bamboo fibers changed from 19 °SR to 79 °SR, there was little change in the fiber length, the spinning degree was low, so its structure stability was good. As a result, when bamboo fibers were added to reconstituted tobacco, the mechanical properties gradually improved. Accordingly, the positive contribution of bamboo fibers beating to the enhancement of mechanical properties outweighs the slight reduction in fiber length and the negative impact caused by the beating degree.

The mechanical properties of reconstituted tobacco declined continuously as the beating degree increased and hardwood fibers were added. Specifically, as the beating degree increased from 20 °SR to 79 °SR, the longitudinal tensile index decreased by 48.91%, the transverse tensile index decreased by 41.98%, and the bursting resistance index declined by 17.53%. This outcome was directly influenced by the inherent properties of hardwood fibers, which have the shortest average fiber length among the five fiber types described in this paper. Additionally, the hardwood fibers composition is complex and includes a variety of cell types, such as ducts, wood fibers, and thin-walled cells of wood rays. This structural diversity may cause a greater susceptibility to damage during the beating process, particularly at higher beating degrees, where fiber breakage and internal structural damage are more pronounced. Furthermore, this reduces the fibers numbers that can effectively contribute to the formation of the web and enhance the structure of the reconstituted tobacco. Ultimately leading to a weakening of its mechanical properties.

The comprehensive analysis founded that the physical strength of reconstituted tobacco improved the most when the (50 ± 1) °SR fibers were added for most fibers. Those results further suggest the reconstituted tobacco enhancement of physical properties by adjusting and controlling the beating degree. Therefore, the evenness, TSO, thermal conductivity, and specific heat capacity of the reconstituted tobacco at around (50 ± 1) °SR were analyzed and tested.

Tensile Strength Indices: TSI_MD_ (Machine Direction Tensile Strength Index); TSI_CD_ (Cross Direction Tensile Strength Index); TSI_MD/CD_ (ratio of longitudinal and transverse tensile strength indices); TSO (Tensile Strength Orientation Angle). TSO is the angle between the maximum tensile strength index and the casting direction (Whelan et al., 2018; Zhang et al., 2018). The maximum expansion of reconstituted tobacco after absorbing water occurred perpendicular to TSO, due to the lateral flow of the slurry in the feed tank (Gao et al., 2014; Niu et al., 2023; Weiyang et al., 2023). Similar to the papermaking process, various production conditions all determine the parameters of SPRT, such as the properties of raw materials, the speed of the carrying tape, the height of the casting line slit, the drying conditions, and so on. These parameters were directly influenced by the orientation of fibers in the reconstituted tobacco (Tamboura et al., 2020; Lee et al., 2022). This study investigated the impact of fiber type on the elasticity of reconstituted tobacco using a controlled variable method (Darlington et al., 1976).

**FIGURE 5:**
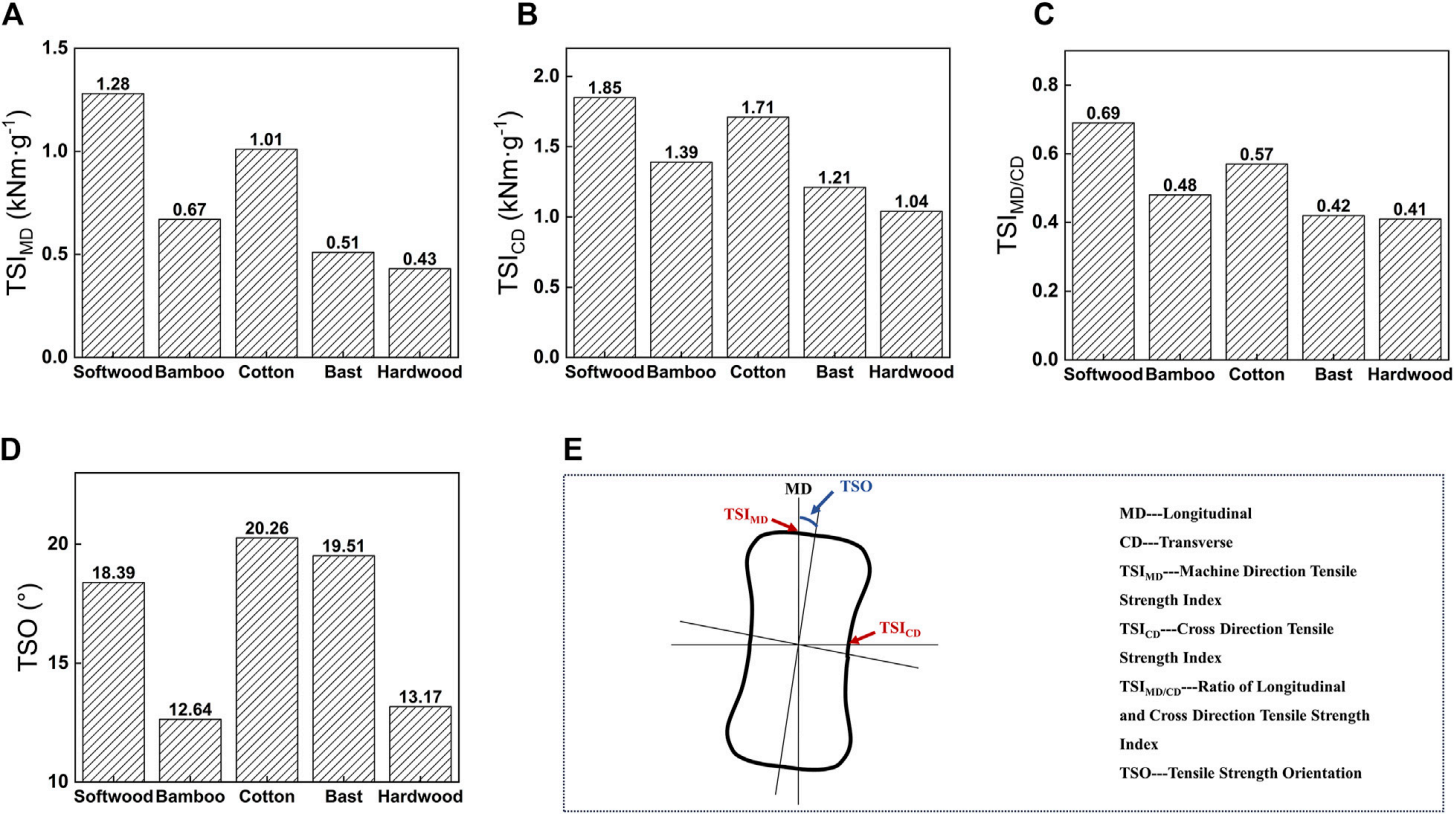
Adding five different kinds of (50 ± 1) °SR fibers to SPRT’s **(A)** TSIMD **(B)** TSICD **(C)** TSIMD/CD **(D)** TSO **(E)** TSO test.

Figure 5 illustrates that when softwood fibers were added to reconstituted tobacco, the TSI_MD_ and TSI_CD_ values were the highest, reaching 1.28 kNm·g⁻^1^ and 1.85 kNm·g⁻^1^. These values aligned with the tensile index characterization results, suggesting that softwood fibers not only enhanced the tensile properties of reconstituted tobacco but also provided optimal elasticity (Baley, 2002; Jiao-Wang et al., 2024). Additionally, the TSO is 18.39°, indicating minimal impact on longitudinal size and good longitudinal stability of the product. On the other hand, reconstituted tobacco with added hardwood fibers shows the lowest TSI_MD_ and TSI_CD_ values, only 0.43 kNm·g⁻^1^ and 1.04 kNm·g⁻^1^. This indicated poor elasticity and a tendency for water expansion and curling deformation due to the sparse dispersion of hardwood fibers in the same proportion. Consequently, the structural stability and uniformity of the reconstituted tobacco were compromised.

The Tensile Strength Orientation Angle (TSO) of the entire sheet directly depended on the consistency of fiber arrangement and its dimensional stability in reconstituted tobacco. In the ideal state, TSO approached 0°, indicating that the direction of the highest tensile strength of the reconstituted tobacco is completely aligned with the direction of thick-pulp casting. This alignment could better meet production demands and reflect the high order of fiber arrangement and the uniformity of the reconstituted tobacco. When comparing the TSO values of five types of reconstituted tobacco (refer to Figure 5D), it was found that the reconstituted tobacco with added bamboo fibers exhibited the smallest TSO angle, measuring only 12.64°. This suggests that bamboo fibers had the most orderly distribution structure in the reconstituted tobacco (Jerpdal et al., 2016; Rittenhouse et al., 2018).

**FIGURE 6:**
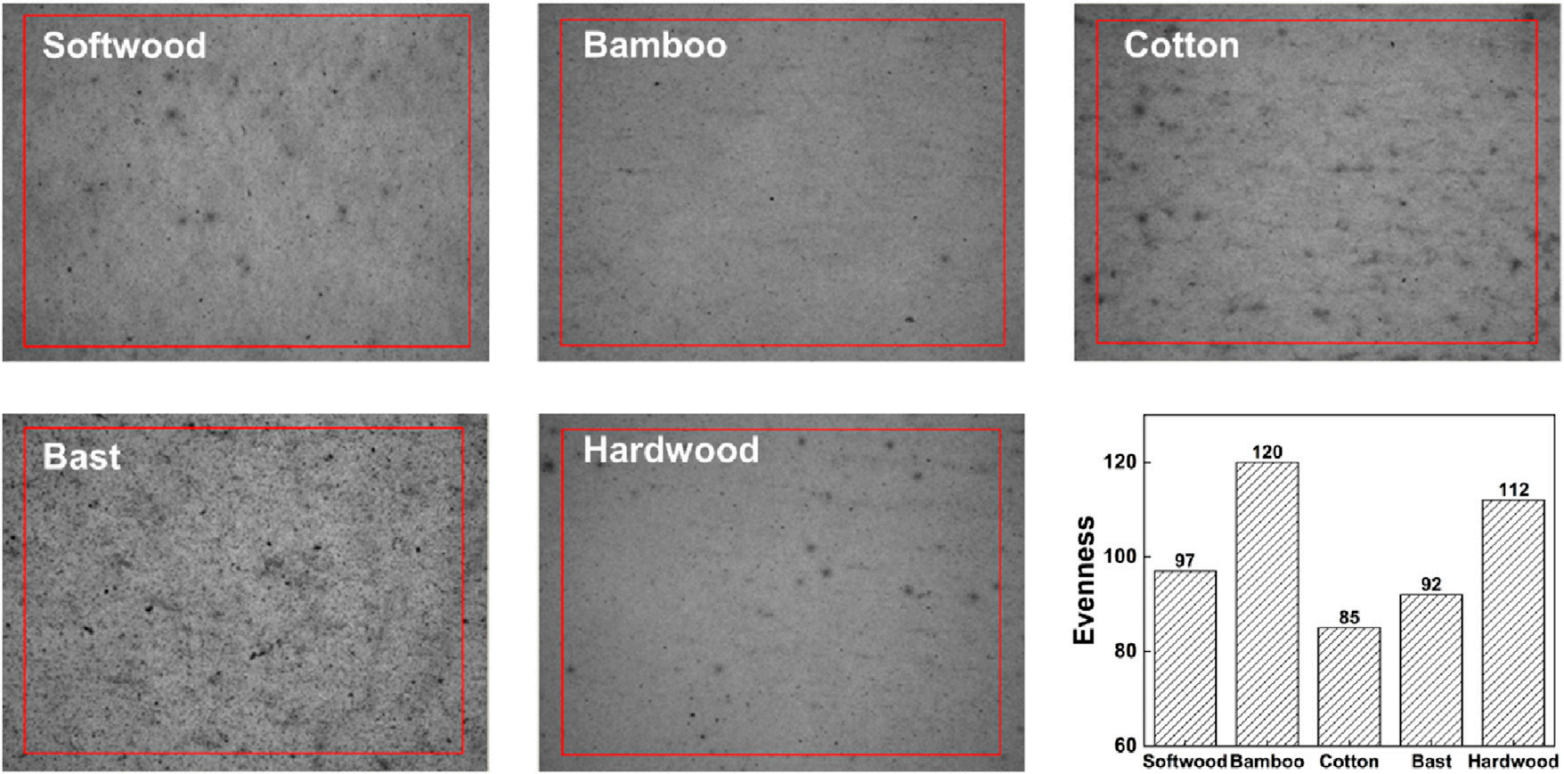
The evenness map and evenness of SPRT with different fibers of (50 ± 1) °SR were added.

The evenness of reconstituted tobacco refers to how evenly the solid components such as fibers and tobacco powder are distributed, including the dispersion of fibers, orientation angle, mode, and the compactness of solid components. This is an important measure of reconstituted tobacco quality and affects its mechanical index values. One reason for reduced evenness is fiber flocculation. The evenness value of reconstituted tobacco produced by the dense pulp method is greatly influenced by the fibers’s characteristics. As shown in Figure 6, among the five types, the evenness index of reconstituted tobacco with added bamboo fibers is the highest at 120, corresponding to the smallest TSO angle. This confirmed that bamboo fibers do not easily clump together, resulting in a uniform and orderly distribution of the reconstituted tobacco. Therefore, adding bamboo fibers could achieve the highest uniformity in reconstituted tobacco.

The thermal conductivity coefficient measures how efficiently a material transfers heat. A higher value indicates better heat transfer for reconstituted tobacco. Research on the thermal conductivity of reconstituted tobacco revealed that the type of added plant fibers affected this property (Chen., et al., 2014; Gao et al., 2015; Guo et al., 2022; Zhao et al., 2024; Zhu et al., 2024). The physical structure and chemical composition of different plant fibers caused changes in the microscopic and macroscopic properties of reconstituted tobacco, such as density, pore distribution, and fiber arrangement. All of these factors together influence the thermal conductivity of reconstituted tobacco (Ding et al., 2017; Dai et al., 2021; Wu et al., 2024; Zhan Y et al., 2024).

**FIGURE 7:**
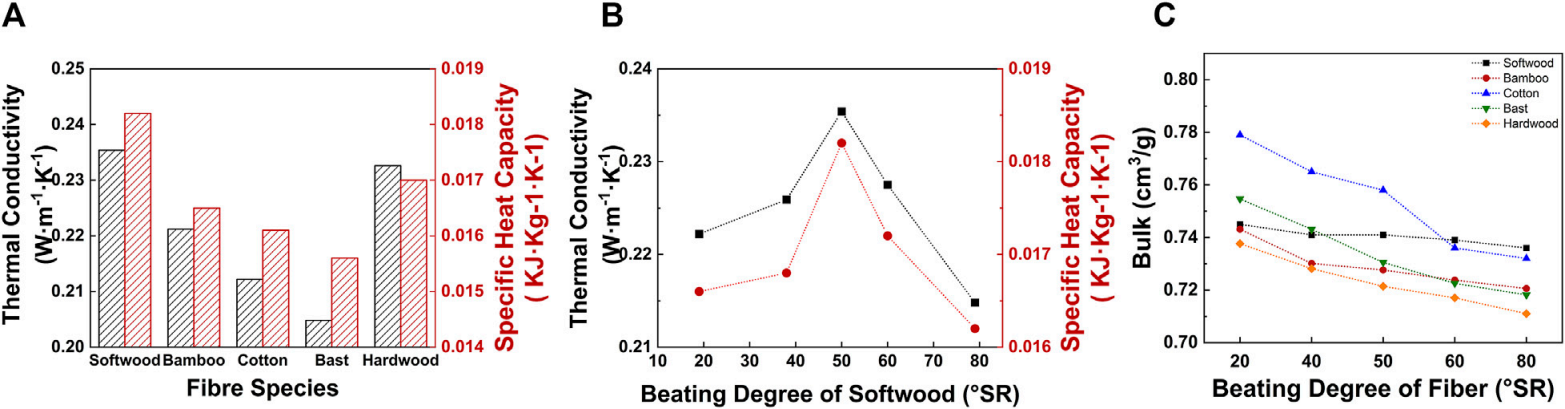
**(A)** Adding five different kinds of (50 ± 1) °SR fibers SPRT **(B)** Adding different beating degree fibers SPRT effect on thermal conductivity and specific heat capacity. **(C)** Adding different beating degree fibers SPRT effect on bulk.

The results displayed in Figure 7A indicate that reconstituted tobacco combined with softwood fibers exhibits the highest thermal conductivity of 0.2354 W·m⁻^1^ K⁻^1^. This advantage could be attributed to the long and fine characteristics of softwood fibers promoting the formation of a connected fibers network within the internal structure. This network constitutes an effective thermal conduction path and reduces the air gap inside the material, thereby enhancing the overall heat transfer efficiency (Chen et al., 2013; Gao et al., 2024).

In addition, the thermal conductivity of reconstituted tobacco was closely related to its loose bulk. Loose bulk (Liu et al., 2019), as an index to characterize the density or porosity of reconstituted tobacco, a large value indicates that the structure is relatively loose and rich in the air (Peng et al., 2021; Gao et al., 2024). Generally speaking, since air is a poor conductor of heat, reconstituted tobacco with high loose bulk has lower thermal conductivity, that is, poor thermal conductivity, compared with reconstituted tobacco with a tight structure, because their porous structure contained a large amount of air gap, which inhibited the transfer of heat flow. As shown in Figure 7C, with the increase of the beating degree of softwood fibers, the loose bulk of the reconstituted tobacco decreased, and the thermal conductivity of the reconstituted tobacco increased with the decrease of the loose bulk before 50 °SR (Zhao et al., 2020). That was when the beating degree was 50 °SR, the thermal conductivity reached the peak value of 0.2354 W·m⁻^1^ K⁻^1^, which increased by 5.94% compared with 0.2222 W·m⁻^1^ K⁻^1^ at 19 °SR. However, when the beating degree continued to increase, the high beating degree led to the increase of the fine components of the fibers and the shortening of the length, resulting in the poor continuity of the network structure formed by the fibers in the reconstituted tobacco (Huang et al., 2021). The negative effect of this factor exceeded the negative impact of the reduction of loose bulk on the improvement of thermal conductivity, and the thermal conductivity of the reconstituted tobacco gradually decreased under the condition of high beating degree.

## 4 Conclusion

In this contribution, we focused on herein the comprehensive properties of SPRT to illustrate the influence rule mechanism of adding plant fibers to reconstituted tobacco. In summary, softwood fibers had the most improvement on the physical properties of reconstituted tobacco, which was mainly attributed to the following reasons: (i) the beating process for wood fibers had a moderate effect on those five kinds of fibers, its length in (0.0–0.05) mm increased from 0.055% to 0.134% after beating 79 °SR, additionally, the average fibers length almost decreased to about half of the original length, from 2.205 mm to 1.241 mm, (ii) the longitudinal tensile, transverse tensile, bursting, and thermal conductivity index of reconstituted tobacco with 50°SR softwood fibers were 7.992 N·m·g⁻^1^, 5.082 N·m·g⁻^1^, 0.271 kPa·m^2^·g⁻^1^, and 0.2354 W·m⁻^1^ K⁻^1^, respectively, which were uppermost of five plant fibers at same beating degree, enhancing by its yarn-spinning to formulate compact fibers network and reduce the air gap inside the material. As illustrated in the proof of principle studies, exploring the mechanism of enhancing the physical properties of reconstituted tobacco with added fibers could not only overcome the shortcomings of insufficient mechanical strength during subsequent processing but also circumvent the intrinsic drawbacks of affecting the consuming experience by causing an unpleasant woody taste. Moreover, this study may provide facile access to the commercial process of HTPs with an extremely maneuverable and effective production rate, promote the industrial production process, and provide a new strategy for large-scale synthesis of a wide range of HTPs.

## Data Availability

The original contributions presented in the study are included in the article/supplementary material, further inquiries can be directed to the corresponding authors.
